# Supplementary material for: Real-time counting of wheezing events from lung sounds using deep learning algorithms: Implications for disease prediction and early intervention
Source: PLoS One. 2023 Nov 20;18(11):e0294447. doi: 10.1371/journal.pone.0294447 (PMC10659186; doi:10.1371/journal.pone.0294447)
Supplement: S1 Table — (DOCX) [file pone.0294447.s001.docx]

| Methodology | Results |
| --- | --- |
| Neural Networks [1] | Assists in automatic detection and classification of adventitious lung sounds. |
| Classifiers [2, 3] | Used for automatic detection and classification of lung sounds. |
| Non-negative Matrix Factorization (NMF) [4] | Assists in automatic detection and classification of adventitious lung sounds. |
| Deep Learning Algorithms [5] | Trains the machine to automatically learn the characteristics of the signals or waveforms of lung sounds. |
| Convolutional Neural Network (CNN) [6-11] | Extracts breathing sound features from a two-dimensional spectrogram image. Accuracy ranges from 63% to 99%. CNN-based model generally has the highest accuracy. |
| Recurrent Neural Network (RNN) Model [12, 13] | Used for lung sound classification. |
| Convolutional-Recurrent Neural Network (CRNN) [5, 14] | Combination of CNN and RNN for lung sound classification. |
| AI-based Lung Sound Analysis [15, 16] | Suggested for determining the degree of airway inflammation or risk of a number of lung diseases. |
| Collection of Breathing Sounds from Smartphones or Real-time Lung Sounds from Wearable Devices [17-22] | Used to develop automated AI-based solutions for lung sound analysis and classification. Through this technological advancement, abnormal respiratory and asthmatic symptoms could be detected or diagnosed at an early stage via real-time self-monitoring or telemedicine. |
| Our study: 1D-CNN and LSTM Network Model | Achieved a detection accuracy of 90%. Not only identifies abnormal sounds within each breath but also captures comprehensive data on their location, duration, and relationships within entire respiratory cycles. Capable of handling continuous data. Utilizes a substantial dataset consisting of 535 respiration cycles from diverse sources. |

**S1 Table. Research trends in lung sound analysis and related papers.**

**References**

1. Kochetov K, Putin E, Azizov S, Skorobogatov I, Filchenkov A, editors. Wheeze detection using convolutional neural networks. Progress in Artificial Intelligence: 18th EPIA Conference on Artificial Intelligence, EPIA 2017, Porto, Portugal, September 5-8, 2017, Proceedings 18; 2017: Springer.

2. Rani A, Sehrawat H, editors. Role Of Machine Learning and Random Forest in Accuracy Enhancement During Asthma Prediction. 2022 10th International Conference on Reliability, Infocom Technologies and Optimization (Trends and Future Directions)(ICRITO); 2022: IEEE.

3. Oletic D, Bilas V. Asthmatic wheeze detection from compressively sensed respiratory sound spectra. IEEE journal of biomedical and health informatics. 2017;22(5):1406-14.

4. Torre-Cruz J, Canadas-Quesada F, Carabias-Orti J, Vera-Candeas P, Ruiz-Reyes N. A novel wheezing detection approach based on constrained non-negative matrix factorization. Applied Acoustics. 2019;148:276-88.

5. Acharya J, Basu A. Deep neural network for respiratory sound classification in wearable devices enabled by patient specific model tuning. IEEE transactions on biomedical circuits and systems. 2020;14(3):535-44.

6. Aykanat M, Kilic O, Kurt B, Saryal S. Classification of lung sounds using convolutional neural networks. EURASIP Journal on Image and Video Processing. 2017;2017(1):1-9.

7. Bardou D, Zhang K, Ahmad SM. Lung sounds classification using convolutional neural networks. Artificial Intelligence in Medicine. 2018;88:58-69.

8. Demir F, Ismael AM, Sengur A. Classification of lung sounds with CNN model using parallel pooling structure. IEEE Access. 2020;8:105376-83.

9. Demir F, Sengur A, Bajaj V. Convolutional neural networks based efficient approach for classification of lung diseases. Health information science and systems. 2020;8(1):1-8.

10. Shuvo SB, Ali SN, Swapnil SI, Hasan T, Bhuiyan MIH. A lightweight cnn model for detecting respiratory diseases from lung auscultation sounds using emd-cwt-based hybrid scalogram. IEEE Journal of Biomedical and Health Informatics. 2020;25(7):2595-603.

11. Tariq Z, Shah SK, Lee Y. Feature-based Fusion using CNN for Lung and Heart Sound Classification. Sensors. 2022;22(4):1521.

12. Kochetov K, Putin E, Balashov M, Filchenkov A, Shalyto A, editors. Noise masking recurrent neural network for respiratory sound classification. International Conference on Artificial Neural Networks; 2018: Springer, Cham.

13. Perna D, Tagarelli A, editors. Deep auscultation: Predicting respiratory anomalies and diseases via recurrent neural networks. 2019 IEEE 32nd International Symposium on Computer-Based Medical Systems (CBMS); 2019: IEEE.

14. Hsu FS, Huang SR, Huang CW, Huang CJ, Cheng YR, Chen CC, et al. Benchmarking of eight recurrent neural network variants for breath phase and adventitious sound detection on a self-developed open-access lung sound database. PLoS One. 2021;16(7):e0254134.

15. Shimoda T, Obase Y, Nagasaka Y, Nakano H, Ishimatsu A, Kishikawa R, et al. Lung sound analysis helps localize airway inflammation in patients with bronchial asthma. Journal of Asthma and Allergy. 2017;10:99-108.

16. Aziz S, Khan MU, Shakeel M, Mushtaq Z, Khan AZ. An Automated System towards Diagnosis of Pneumonia using Pulmonary Auscultations. 2019 13th International Conference on Mathematics, Actuarial Science, Computer Science and Statistics (MACS)2019. p. 1-7, doi: 10.1109/MACS48846.2019.9024789.

17. Rocha BM, Filos D, Mendes L, Serbes G, Ulukaya S, Kahya YP, et al. An open access database for the evaluation of respiratory sound classification algorithms. Physiological Measurement. 2019;40(3):035001.

18. Faezipour M, Abuzneid A. Smartphone-based self-testing of COVID-19 using breathing sounds. Telemedicine and e-Health. 2020;26(10):1202-5.

19. Gupta P, Moghimi MJ, Jeong Y, Gupta D, Inan OT, Ayazi F. Precision wearable accelerometer contact microphones for longitudinal monitoring of mechano-acoustic cardiopulmonary signals. NPJ Digital Medicine. 2020;3(1):1-8.

20. Jaber MM, Abd SK, Shakeel PM, Burhanuddin MA, Mohammed MA, Yussof S. A telemedicine tool framework for lung sounds classification using ensemble classifier algorithms. Measurement. 2020;162(107883).

21. Srivastava A, Jain S, Miranda R, Patil S, Pandya S, Kotecha K. Deep learning based respiratory sound analysis for detection of chronic obstructive pulmonary disease. PeerJ Computer Science. 2021;7:e369.

22. Lella KK, Pja A. Automatic diagnosis of COVID-19 disease using deep convolutional neural network with multi-feature channel from respiratory sound data: cough, voice, and breath. Alexandria Engineering Journal. 2022;61(2):1319-34.
