# Supplementary material for: Real-time counting of wheezing events from lung sounds using deep learning algorithms: Implications for disease prediction and early intervention
Source: PLoS One. 2023 Nov 20;18(11):e0294447. doi: 10.1371/journal.pone.0294447 (PMC10659186; doi:10.1371/journal.pone.0294447)
Supplement: S2 Table — (DOCX) [file pone.0294447.s002.docx]

| **Layer** | **Filters** | **Kernel/ pool size** | **Units** | **Number of**  **Parameters** | **Activation function** |
| --- | --- | --- | --- | --- | --- |
| **Conv 1d** | 32 | 16 | -- | 544 | ReLu |
| **Conv 1d** | 32 | 16 | -- | 16416 | ReLu |
| **MaxPooling 1D** | -- | 2 | -- | -- |  |
| **Bidirectional LSTM** | -- | -- | 256 | 591872 |  |
| **Dense** | -- | -- | 128 | 65664 | ReLu |
| **Dense** | -- | -- | 3 | 387 | SoftMax |

**S4 Table. Model parameter values of the 1D CNN + LSTM model.**
