# Supplementary figures and images for: Real-time counting of wheezing events from lung sounds using deep learning algorithms: Implications for disease prediction and early intervention
Source: PLoS One. 2023 Nov 20;18(11):e0294447. doi: 10.1371/journal.pone.0294447 (PMC10659186; doi:10.1371/journal.pone.0294447)

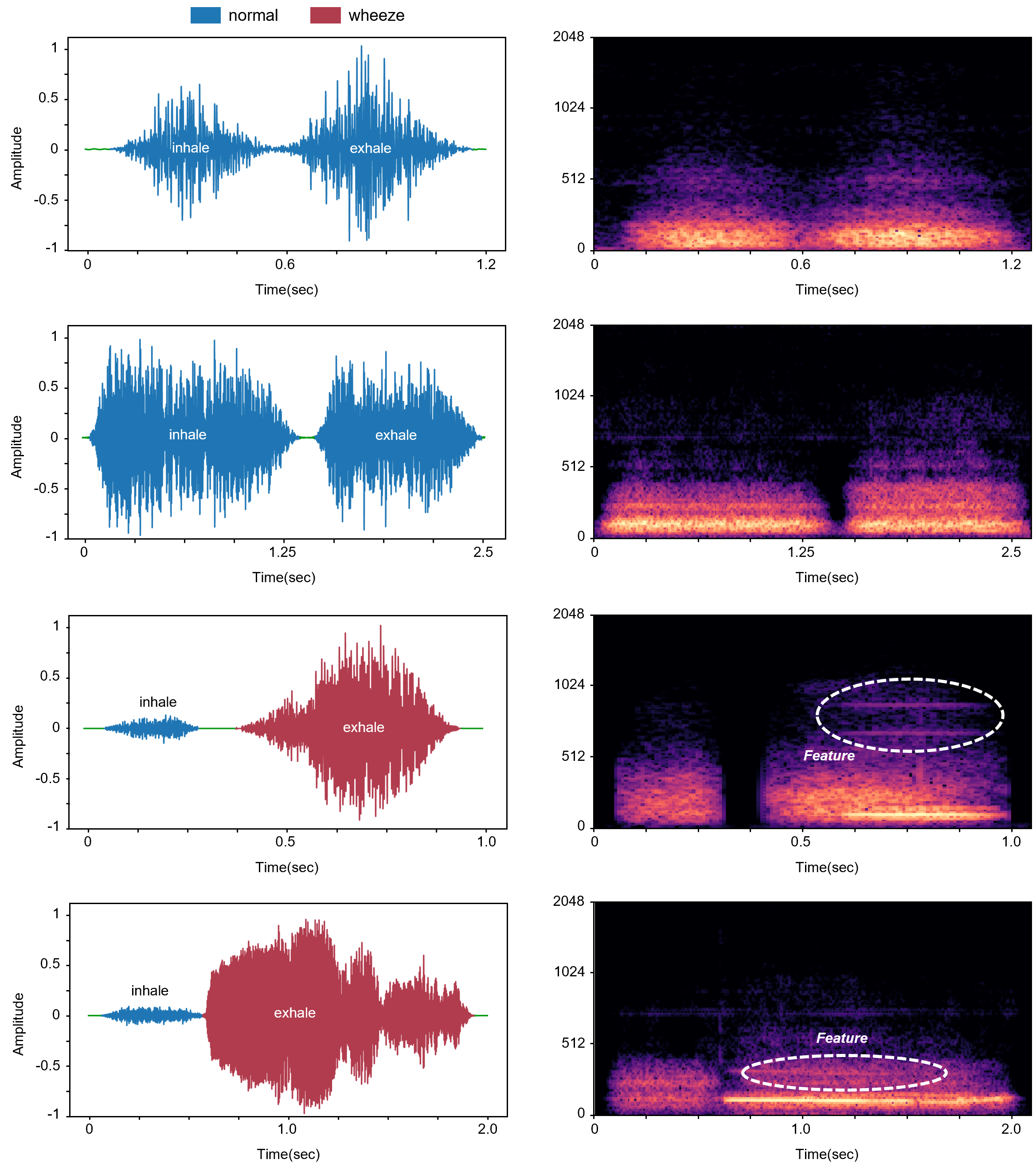

Supplement: S1 Fig — In some cases, there is coexistence of normal and wheeze sound in isolated breathing cycle. (TIF) [file pone.0294447.s003.tif]

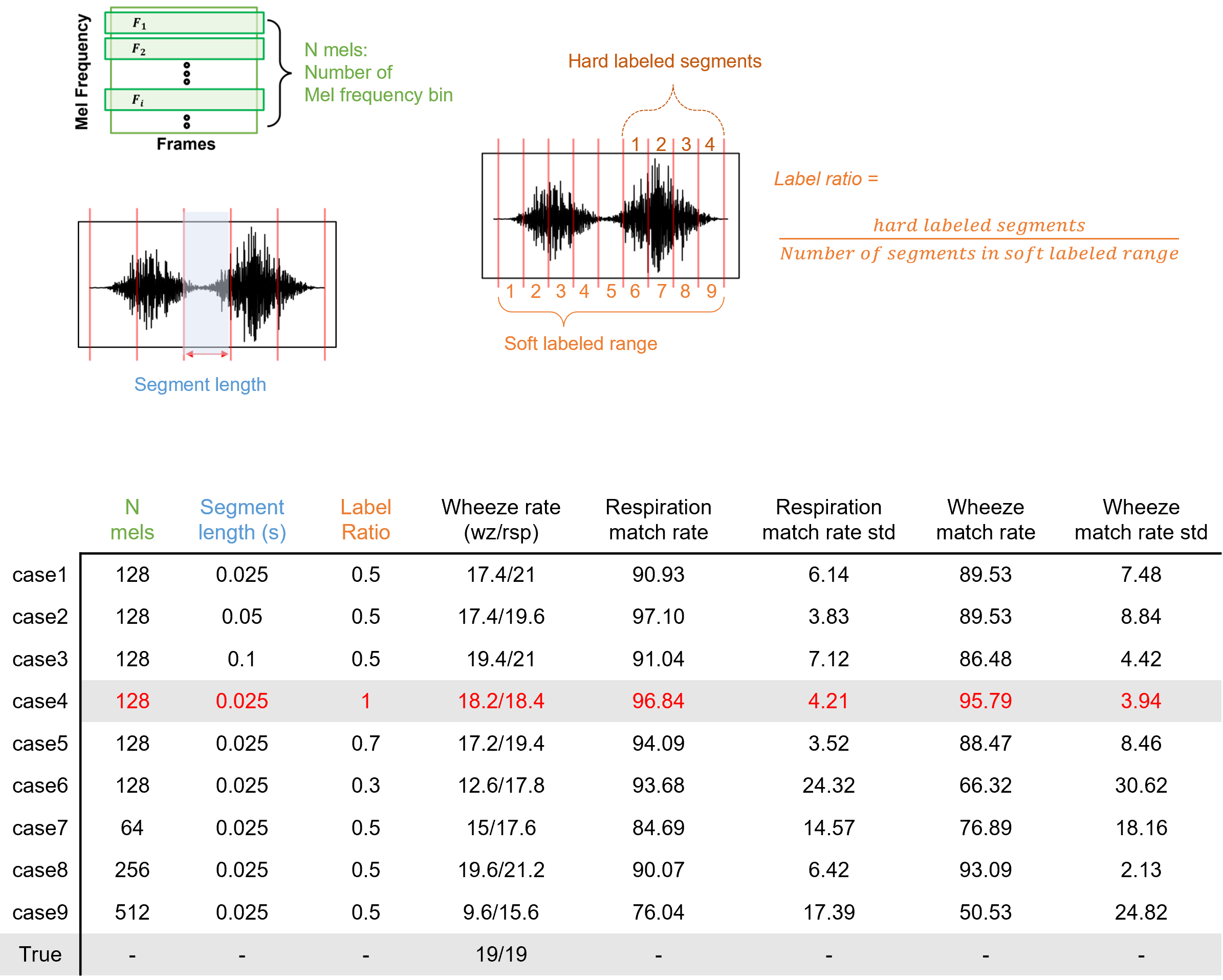

Supplement: S2 Fig — Among 9 cases of parametric study, we choose parameters of case 4 to utilize in counting algorithm. (TIF) [file pone.0294447.s004.tif]

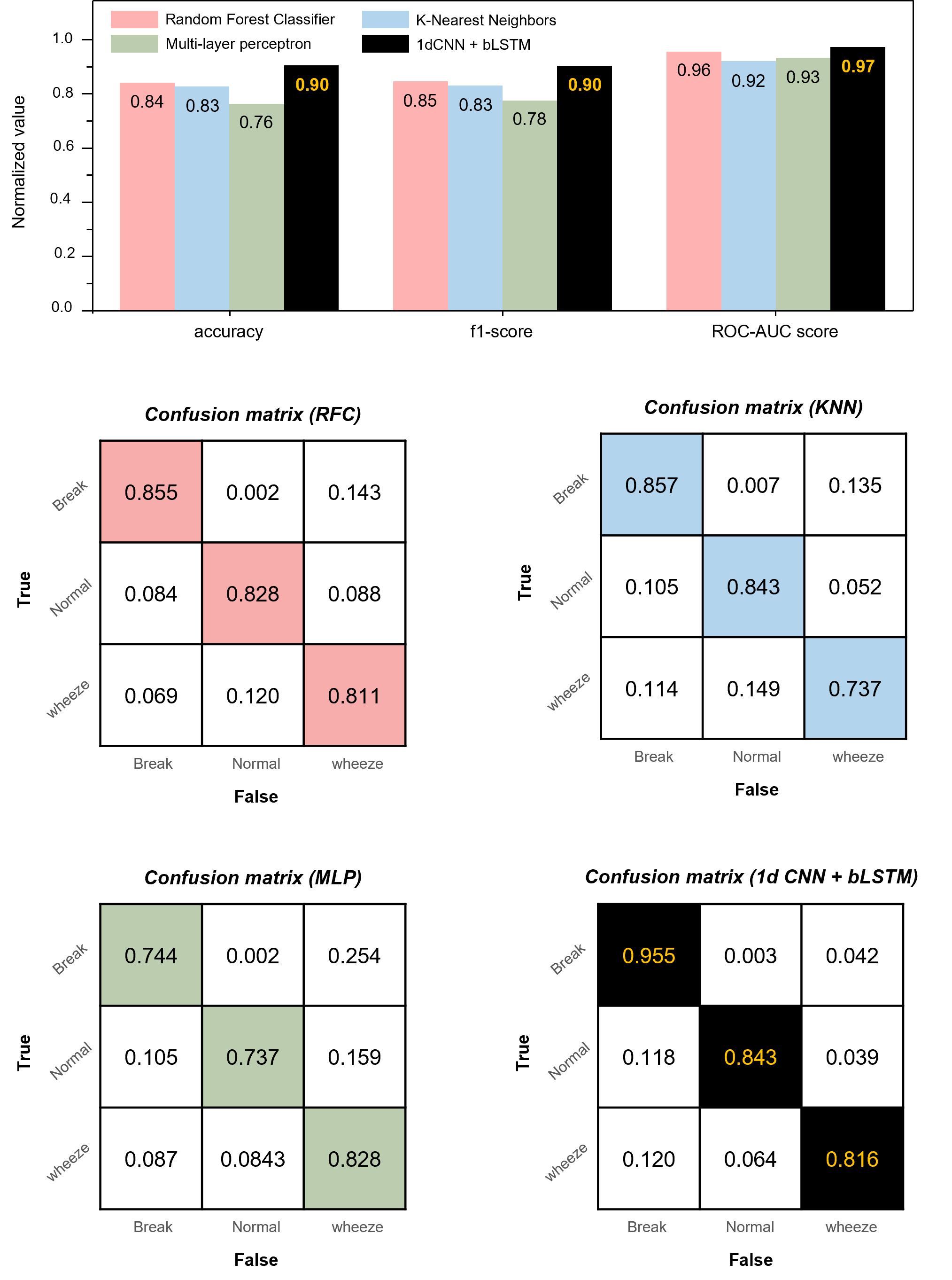

Supplement: S3 Fig — The classifier also trained by 10-fold cross-validation method. (TIF) [file pone.0294447.s005.tif]

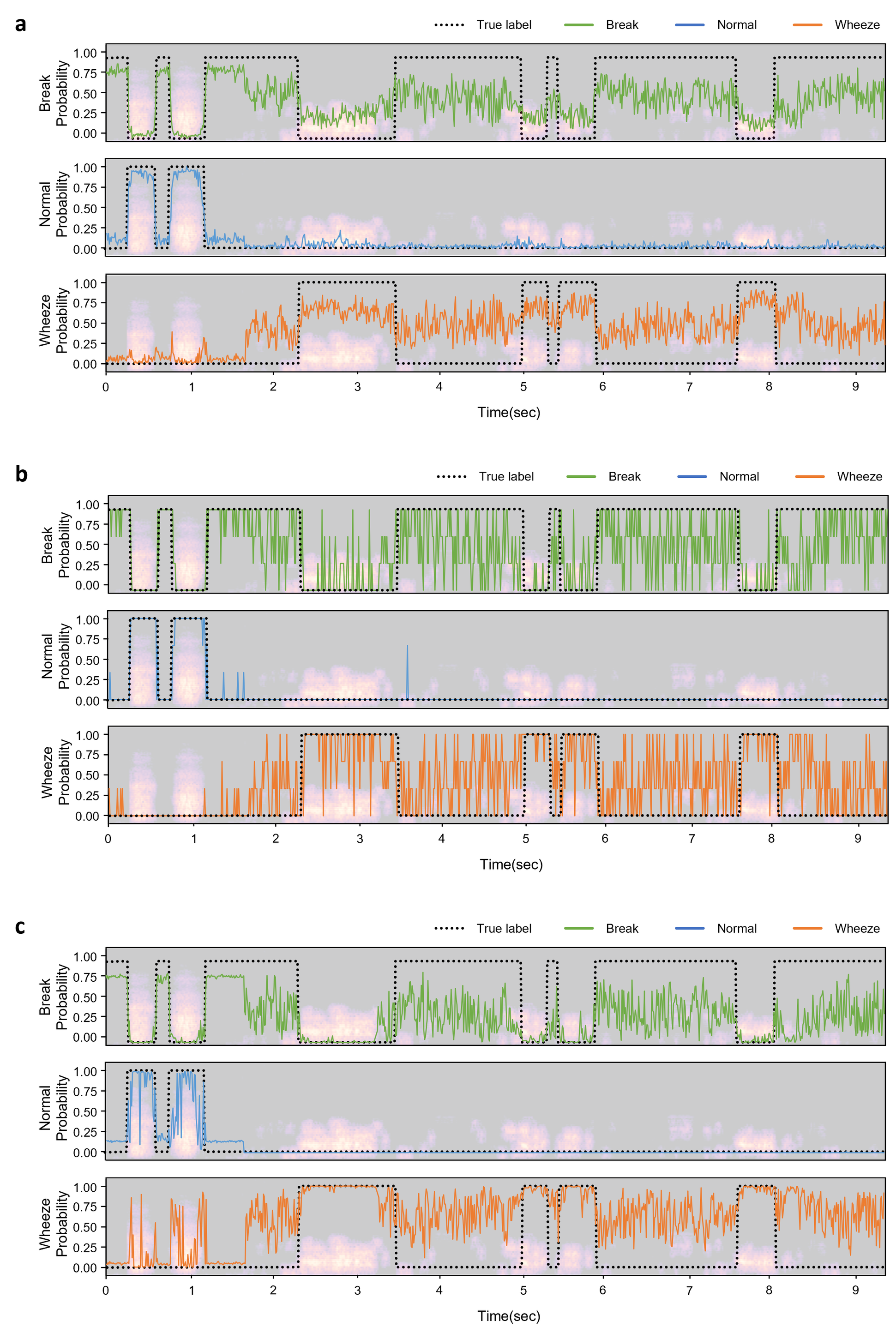

Supplement: S4 Fig — (A) Random Forest classifier, (B) K-Nearest Neighbors, (C) Multi-layer perceptron. (TIF) [file pone.0294447.s006.tif]

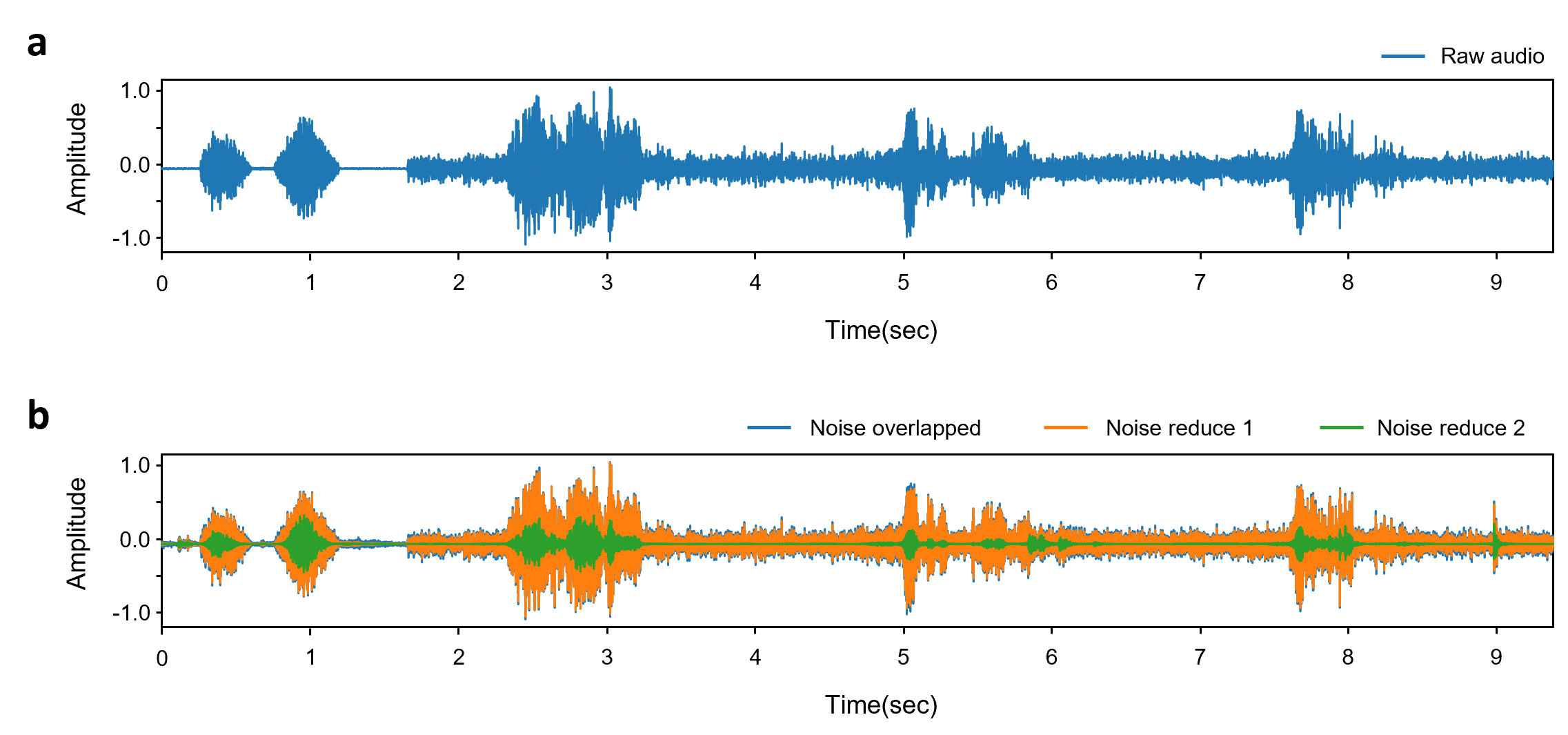

Supplement: S5 Fig — (A) original raw signal of test data, (B) test data with overlapped noise (SNR -20dB) is depicted in blue line, and result after noise reduce is plotted in orange line (The number of standard deviations above the noise is set to ‘0.1’, and mode of stationary set to ‘True’), and green line (default setting from library). (TIF) [file pone.0294447.s007.tif]

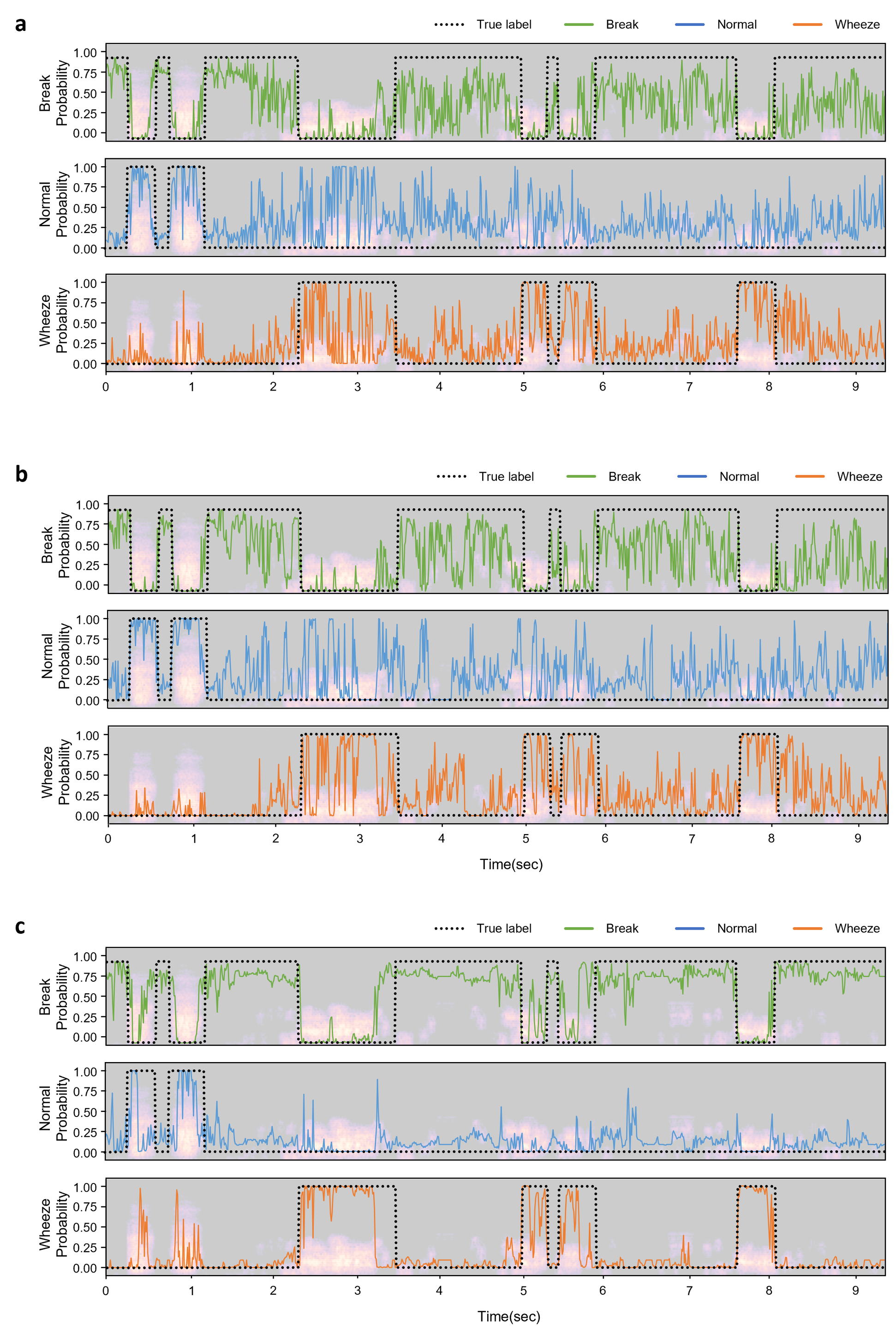

Supplement: S6 Fig — (A) prediction probabilities of original noisy data, (B) predictions of noise reduced data (The number of standard deviations above the noise is set to ‘0.1’, and mode of stationary set to ‘True’), and (C) different setting of noise reduced data (default setting from library). (TIF) [file pone.0294447.s008.tif]
