# Supplementary material for: Real-time counting of wheezing events from lung sounds using deep learning algorithms: Implications for disease prediction and early intervention
Source: PLoS One. 2023 Nov 20;18(11):e0294447. doi: 10.1371/journal.pone.0294447 (PMC10659186; doi:10.1371/journal.pone.0294447)
Supplement: Attachment — Submitted filename: Response to Reviewers.docx [file pone.0294447.s010.docx]

|   **Daeshik Kang, PhD**  Ajou University professor  Department of  mechanical engineering  Tel: +82-31-219-2345  [dskang@ajou.ac.kr](mailto:dskang@ajou.ac.kr) |   **Dohyeong Kim, PhD**  University of Texas at Dallas  Professor  School of Economic, Political and Policy Sciences  Tel: +1-972-883-3512  [dohyeong.kim@utdallas.edu](mailto:dohyeong.kim@utdallas.edu) |   **Sungchul Seo, PhD**  Seokyeong University professor  Department of Nano-Chemical,  Biological and  Environmental Engineering  Tel: +82-2-940-2914  [haha0694@gmail.com](mailto:haha0694@gmail.com) |
| --- | --- | --- |

3 August 2023

Dear Editors,

We appreciate for recent correspondence regarding our manuscript entitled “Real-time counting of wheezing events from lung sounds using deep learning algorithms: implications for disease prediction and early intervention” by S. Im et al. We appreciate the thoughtful and insightful comments provided by the reviewers, which have helped us to further improve the contents of our research. In the following pages, we have addressed each of the reviewers' comments, along with our responses and the associated modifications, which have been highlighted in the manuscript.

Based on the reviewers' attentive comments, we believe that the revised manuscript is now suitable for publication in *PLOS ONE*. We would like to express our gratitude to the reviewers for their time and attention in helping us to enhance the quality of our work.

Sincerely,

Daeshik Kang

**Response to Reviewer 1**

**General comments:**
The paper proposes to use the 1d-cnn to count the wheeze events from lung sound dataset.
The problem of study is interesting and relevant.
However, the paper requires several issues to be fixed for the publication.

We appreciate you to accepting the definition of the issues. We spent plenty of time debating how to respond to your insightful comments, and we responded by highlighting each one with blue. Additionally, we highlighted our revised manuscript with red. We hope that our responses sufficiently satisfy your comments.

**Comment 1**:
The graphics are poor, which is difficult to understand.

**Our response:**
We would like to express our appreciation to the reviewer for providing intuitive feedback. We checked suggested papers in your comment (2) and find intuitive diagram of designed model. We rearrange the graphics to our Figure 3.

| **Before:**   | **After:**   |
| --- | --- |

**Comment 2:**
Authors are suggested discussing and citing some state of the art sound classification papers published in top journals.
<https://ieeexplore.ieee.org/abstract/document/9784899>
<https://ieeexplore.ieee.org/abstract/document/9931407>
<https://ieeexplore.ieee.org/abstract/document/9684869>
These papers explain the cnn-lstm, mfcc, ensemble learning for the different kinds of audio sound (eg. lung sound, etc.) classification, which are closely related to the current study.

**Our response:**
We would like to express our appreciation to the reviewer for providing valuable feedback. As you mentioned, suggested papers are somewhat in the line with our research. Among the papers, two papers are about evaluating neonatal bowel sound, and it is very interesting that the bowel sound could be utilized as vital cue for infant care. Our research is, however, about lung sound monitoring by counting the frequency of wheezing evoked in COPD or Asthma patient, explored to environmental affect about respiratory diseases. Othe other hand, the other paper suggested is about real-time lung sound quality assessment for telehealth applications, so we decided to cite the paper of lung sound quality assessment in our discussion section where we discuss our research’s limitation and future work.

| **Before:**  First, due to the limited availability of reference lung sound data collected in good quality, it is not sufficiently verified whether the developed algorithm would function well on the data collected from various patients in diverse recording environments. The algorithm must be further enhanced and adjusted based on the clinical trials of long-term lung sound monitoring with a broad patient group in order to assure the validity, reliability, and applicability for preventive treatment in clinical and non-clinical settings. | **After:**  First, due to the limited availability of reference lung sound data collected in good quality, it is not sufficiently verified whether the developed algorithm would function well on the data collected from various patients in diverse recording environments. With useful method such as assessment of lung sound quality [1], we would utilize fine quality of lung sound data resulting in more sophisticated and accurate AI model. Furthermore, the algorithm must be enhanced and adjusted based on the clinical trials of long-term lung sound monitoring with a broad patient group in order to assure the validity, reliability, and applicability for preventive treatment in clinical and non-clinical settings.  1. Grooby E, Sitaula C, Fattahi D, Sameni R, Tan K, Zhou L, et al. Real-time multi-level neonatal heart and lung sound quality assessment for telehealth applications. IEEE Access. 2022;10:10934-48. |
| --- | --- |

**Comment 3:**
It is unclear how the current study is applicable unlike previously proposed model in the field.

**Our response:**
We thank to your comment to explain our research in detail. Our paper suggests the counting algorithm of the wheeze sounds. We didn’t mean to suggest new model of classification, but the applicable method for practical using by our proposed algorithm. We use the predictions of segment-based AI model such as CNN-LSTM combined model which we used in our research and interpret the results of prediction to monitor the frequency and timing of wheeze sounds. We didn’t insist that our model is more suitable than other models, but we suggest the applicable methods to use AI models. It is important point to convince post-readers of our paper, so we added more clear explanation about what we did in the paper at discussion section.

| **Before:**  Otherwise, we present a method for implementing long-term monitoring in clinical or non-clinical settings that counts the number of wheeze occurrences over time. Our algorithm utilizes segment-based classification, which is normally used in speech recognition [2] or rare sound detection [3, 4]. | **After:**  Otherwise, we present an applicable method for implementing long-term monitoring in clinical settings by counting the number of wheeze occurrences over time. Our method differs from previous research in that it counts both the number of normal breaths and the number of wheezes, that is helpful for monitoring respiratory disease patients in dynamic environments. We utilizes segment-based classification AI model, which is normally used in speech recognition [2] or rare sound detection [3, 4]. |
| --- | --- |

**Comment 4:**
The paper requires to perform the comparative study with the state of the art methods, which could establish trustworthiness.

**Our response:**
We thank to your review, we additionally compared several models from ensemble, decision tree which are applicable to our counting algorithm. The results are summarized in Supplementary Figure 4.

| **Before:**  Additionally, prior to applying the trained model to the counting algorithm, we contrasted it with a simple SVM classifier example (S4 Fig). To further accurately assess the performance of the two models, we used identical test datasets for each. | **After:**  Additionally, prior to applying the trained model to the counting algorithm, we also compared it to three popular and verified classifiers from Si-kit Learn library: Random Forest Classifier, K-Nearest Neighbors, and Multi-layer Perceptron classifier (S4 Fig, S5 Fig). To further accurately assess the performance of those models, we used identical test datasets for each. In every indicator, the results demonstrated that the 1dCNN + bLSTM model performed better than the others. Because of this, we used the model to implement our algorithm. |
| --- | --- |
| **Before:**   | **After:**   |

**Comment 5:**
The dataset is small, please use larger datasets.

**Our response:**
Thanks for intuitive comment. As so, we additionally added ICBHI 2017 challenge datasets to convince our counting algorithm. Among the datasets, we used annotated wheeze signals.

| **Before:**  We obtained reference lung sound signals from three databases: 1) lung sound simulator, 2) EMTprep, and 3) clinical patient records. First, using a commercial microphone, ten and seven cycles of typical breathing and wheezing data were taken from the pediatric lung sound simulator known as Child Sim (SimulAIDS Inc, UK). Second, the open-source lung sound database EMTprep (https://www.EMTprep.com) provided three cycles of typical breathing and nine cycles of wheezing sound data. And last, we used 17 cycles of wheeze breathing using a commercial microphone that was affixed to the anterior right lung region. | **After:**  We obtained reference lung sound signals from three databases: 1) lung sound simulator, 2) EMTprep, 3) clinical patient records, and 4) ICBHI 2017 datasets. First, using a commercial microphone, ten and seven cycles of typical breathing and wheezing data were taken from the pediatric lung sound simulator known as Child Sim (SimulAIDS Inc, UK). Second, the open-source lung sound database EMTprep (https://www.EMTprep.com) provided three cycles of typical breathing and nine cycles of wheezing sound data. In the case of clinical data, we used 17 cycles of wheeze breathing using a commercial microphone that was affixed to the anterior right lung region. And last, we utilized additional diagnostic data from the ICBHI 2017 challenge database[5]. In the database, we used cases of asthma, COPD, and healthy patients.  5. Rocha BM, Filos D, Mendes L, Serbes G, Ulukaya S, Kahya YP, et al. An open access database for the evaluation of respiratory sound classification algorithms. Physiological measurement. 2019;40(3):035001. |
| --- | --- |
| **Before:** **Type** \| **Simulator** \| **EMTprep** \| **Clinical** \| **Total**; **Normal respiration** \| 10 \| 3 \| -- \| 13; **Wheeze respiration** \| 7 \| 9 \| 17 \| 33 **Label** \| **Simulator** \| **EMTprep** \| **Clinical** \| **Total**; **Break (0)** \| 899 \| 399 \| -- \| 1298; **Normal (1)** \| 699 \| 708 \| -- \| 1407; **Wheeze (2)** \| 159 \| 1144 \| 1844 \| 3147 | |
| **After:** **Type** \| **Simulator** \| **EMTprep** \| **Clinical** \| **ICBHI** \| **Total**; **Normal respiration** \| 10 \| 3 \| -- \| 287 \| 300; **Wheeze respiration** \| 7 \| 9 \| 17 \| 202 \| 235 **Label** \| **Simulator** \| **EMTprep** \| **Clinical** \| **ICBHI** \| **Total**; **Break (0)** \| 899 \| 399 \| -- \| 253,998 \| 255,296; **Normal (1)** \| 699 \| 708 \| -- \| 43,089 \| 44,496; **Wheeze (2)** \| 159 \| 1144 \| 1844 \| 29,781 \| 32,928 | |

**Comment 6:**
The paper requires statistical significance test.

**Our response:**
Thank you for your valuable comment, but our research doesn’t have any data that would make a statistical significance test required. Instead, we compared different AI models based on how well they classified segmented based prediction of the test data. Supplementary Figure 4 provides a summary of the results. (Please refer to the updates provided in the response to your comment 4.)

**Response to Reviewer #2**

**General comments:**
This is an interesting paper where authors propose a real-time wheezing sound counting algorithm based on a one-dimensional convolutional neural network and a long short-term memory combined (1D-CNN-LSTM) network model. In general, the paper is well-written and technically sound, and addresses a problem that may be of high-interest to the biomedical signal processing community. However, the opinion of this reviewer is that the following concerns need to be carefully resolved before resubmission.

We appreciate you to accepting the definition of the issues and suggest valuable comments. We spent plenty of time debating how to respond to your insightful and constructive comments. We responded by highlighting each one with blue. And additionally, we highlighted our revised manuscript with red. We hope that our responses sufficiently satisfy your comments.

**Comment 1:**
First, in the Introduction, the authors present the state of the art in automatic detection and classification of adventitious lung sounds (second and third paragraphs of the section 1), but I think it is not complete, since some important references are missing. For example, in the last decade, algorithms based on non-negative matrix factorization (NMF) have been widely used in the detection-classification of wheezing sound.

**Our response:**
We would like to express our appreciation to the reviewer for providing valuable feedback. As you mentioned, suggested method of algorithm NMF and related research is quite worth mentioning in introduction. So, we decided to cite paper about NMF in our introduction.

| **Before:**  To overcome the limitation of conventional auscultation, various deep learning and sound analysis algorithms, such as automatic detection and classification of adventitious lung sounds, have been developed [6]. Among them, deep learning These algorithms train the machine to automatically learn the characteristics of the signals or waveforms of lung sounds to recognize abnormal lung or breathing sounds (wheezing, crackling) [7]. The most common algorithm used for lung sound classification is a convolutional neural network (CNN) [8-11] or recurrent neural network (RNN) model [12, 13] that extracts breathing sound features from a two-dimensional spectrogram image, or a combination of the two, a convolutional-recurrent neural network (CRNN) [7, 14]. | **After:**  To overcome the limitation of conventional auscultation, various methods such as neural networks [15], classifiers [16, 17], and NMF [18] are suggested in many literatures in order to assist automatic detection and classification of adventitious lung sounds [6]. Among them, deep learning algorithms train the machine to automatically learn the characteristics of the signals or waveforms of lung sounds to recognize abnormal lung or breathing sounds (wheezing, crackling) [7]. The most common deep learning algorithm used for lung sound classification is a convolutional neural network (CNN) [8-11] or recurrent neural network (RNN) model [12, 13] that extracts breathing sound features from a two-dimensional spectrogram image, or a combination of the two, a convolutional-recurrent neural network (CRNN) [7, 14].  15. Kochetov K, Putin E, Azizov S, Skorobogatov I, Filchenkov A, editors. Wheeze detection using convolutional neural networks. Progress in Artificial Intelligence: 18th EPIA Conference on Artificial Intelligence, EPIA 2017, Porto, Portugal, September 5-8, 2017, Proceedings 18; 2017: Springer.  16. Rani A, Sehrawat H, editors. Role Of Machine Learning and Random Forest in Accuracy Enhancement During Asthma Prediction. 2022 10th International Conference on Reliability, Infocom Technologies and Optimization (Trends and Future Directions)(ICRITO); 2022: IEEE.  17. Oletic D, Bilas V. Asthmatic wheeze detection from compressively sensed respiratory sound spectra. IEEE journal of biomedical and health informatics. 2017;22(5):1406-14.  18. Torre-Cruz J, Canadas-Quesada F, Carabias-Orti J, Vera-Candeas P, Ruiz-Reyes N. A novel wheezing detection approach based on constrained non-negative matrix factorization. Applied Acoustics. 2019;148:276-88. |
| --- | --- |

**Comment 2:**
I strongly believe that the database used by the authors is rather limited. Specifically, they have constructed a database consisting of 46 respiratory cycles (13 normal respiration/33 wheezing respiration). Then using data augmentation techniques, they have obtained a total of 5852 respiratory segments of 25 ms labelled as normal, wheeze or break. In this sense, my main concern is whether the dataset is sufficiently representative (has enough variability) to validate the performance of the proposed algorithm. In line 335 of the manuscript, the authors note the limited availability of reference lung sounds. However, in recent years “the publicly available ICBHI 2017 Challenge dataset [A]” has been widely used to assess the performance of adventitious sound detection/classification algorithms. I strongly encourage authors to use this database or a part of it to complete the database proposed by the authors.

[A] ICBHI 2017 challenge, respiratory sound database, 2017,
<https://bhichallenge.med.auth.gr/ICBHI_2017_Challenge>

**Our response:**
Thanks for intuitive comment. As so, we additionally added ICBHI 2017 challenge datasets to convince our counting algorithm. Among the datasets, we chose data from Asthma and COPD patients and healthy patients. As a result, we could train the model with 10 times bigger than prior datasets.

| **Before:**  We obtained reference lung sound signals from three databases: 1) lung sound simulator, 2) EMTprep, and 3) clinical patient records. First, using a commercial microphone, ten and seven cycles of typical breathing and wheezing data were taken from the pediatric lung sound simulator known as Child Sim (SimulAIDS Inc, UK). Second, the open-source lung sound database EMTprep (https://www.EMTprep.com) provided three cycles of typical breathing and nine cycles of wheezing sound data. And last, we used 17 cycles of wheeze breathing using a commercial microphone that was affixed to the anterior right lung region. | **After:**  We obtained reference lung sound signals from three databases: 1) lung sound simulator, 2) EMTprep, 3) clinical patient records, and 4) ICBHI 2017 datasets. First, using a commercial microphone, ten and seven cycles of typical breathing and wheezing data were taken from the pediatric lung sound simulator known as Child Sim (SimulAIDS Inc, UK). Second, the open-source lung sound database EMTprep (https://www.EMTprep.com) provided three cycles of typical breathing and nine cycles of wheezing sound data. In the case of clinical data, we used 17 cycles of wheeze breathing using a commercial microphone that was affixed to the anterior right lung region. And last, we utilized additional diagnostic data from the ICBHI 2017 challenge database[5]. In the database, we used cases of asthma, COPD, and healthy patients.  5. Rocha BM, Filos D, Mendes L, Serbes G, Ulukaya S, Kahya YP, et al. An open access database for the evaluation of respiratory sound classification algorithms. Physiological measurement. 2019;40(3):035001. |
| --- | --- |
| **Before:** **Type** \| **Simulator** \| **EMTprep** \| **Clinical** \| **Total**; **Normal respiration** \| 10 \| 3 \| -- \| 13; **Wheeze respiration** \| 7 \| 9 \| 17 \| 33 **Label** \| **Simulator** \| **EMTprep** \| **Clinical** \| **Total**; **Break (0)** \| 899 \| 399 \| -- \| 1298; **Normal (1)** \| 699 \| 708 \| -- \| 1407; **Wheeze (2)** \| 159 \| 1144 \| 1844 \| 3147 | |
| **After:** **Type** \| **Simulator** \| **EMTprep** \| **Clinical** \| **ICBHI** \| **Total**; **Normal respiration** \| 10 \| 3 \| -- \| 287 \| 300; **Wheeze respiration** \| 7 \| 9 \| 17 \| 202 \| 235 **Label** \| **Simulator** \| **EMTprep** \| **Clinical** \| **ICBHI** \| **Total**; **Break (0)** \| 899 \| 399 \| -- \| 253,998 \| 255,296; **Normal (1)** \| 699 \| 708 \| -- \| 43,089 \| 44,496; **Wheeze (2)** \| 159 \| 1144 \| 1844 \| 29,781 \| 32,928 | |

**Comment 3:**
On the other hand, the training and validation model proposed by the authors is often not used to evaluate the performance of algorithms due to its limitations. The authors simply divide the dataset into train and test. But, this validation methodology is highly dependent on the data used in both groups. That is, the performance obtained can vary significantly when the train and test group is composed of other data. In this sense, I recommend the authors to use a cross-validation methodology to reduce the variance of the estimated performance metrics. For example, a 10-fold cross-validation methodology.

**Our response:**
Thanks for intuitive and constructive feedback. I’d like to explain clearer about our evaluation process. As you suggested we trained the model with 10-fold cross-validation method. Then, among the trained model, we chose the model of highest accuracy. And for evaluation, we used totally un-seen test data, that is not used during the training process.

| **Before:**  The hard-labeled dataset was divided into train and test data. 70% of the data were utilized for the training-validation procedure, and the remaining 30% were used to test the trained model. Among training-validation data, training and validation data were divided into training datasets and validation datasets in a 7:3 ratio. We evaluated the trained model's performance using test data by calculating its Accuracy score, F1 score, and ROC-AUC (Area Under Receiver Operating Characteristic Curve) score. | **After:**  The hard-labeled dataset was divided into train and validation data. We used the 10-fold cross-validation method. We utilized the model with the highest accuracy from among the trained models. Then we evaluated the trained model's performance using test data by calculating its Accuracy score, F1 score, and ROC-AUC (Area Under Receiver Operating Characteristic Curve) score. The test data is extracted from reference audio signals, which are totally unseen during the training process (Fig 5A). |
| --- | --- |

**Comment 4:**
Finally, the authors do not mention how the proposed algorithm performs in noisy environments. In my opinion, I think it would be quite interesting for the authors to show the performance obtained by the proposed algorithm when the analyzed lung sounds are overlapped with real clinical sounds. Authors can find clinical sounds recorded in real environments available online, for example: <https://www.soundsnap.com/tags/clinic>

**Our response:**
Thank you for your considerate review to upgrade our research. As your comment, we tested our algorithm when hospital noise is overlapped.

| **Before:**  None | **After:**  Furthermore, in order to evaluate its durability in noisy environments, we also overlapped clinical environment noise from free open source (https://freesound.org/people/bassboybg/sounds/201638/, S6 Fig). to the test data. As shown in S7 Fig, the model misidentified the noise sound as wheezing, making it difficult to count the number of wheezing. However, after noisereduce Python library preprocessing, the model closely identified the true labels of the test data with less error. These findings show possible effectiveness in noisy environment when the model is combined with proper noise cancellation method. |
| --- | --- |
| **Before:**  None | |
| **After:**    **S6 Fig. Test data overlapped with hospital noise (waiting room).** (A) original raw signal of test data, (B) test data with overlapped noise (SNR -20dB) is depicted in blue line, and result after noise reduce is plotted in orange line (The number of standard deviations above the noise is set to ‘0.1’, and mode of stationary set to ‘True’), and green line (default setting from library).    **S7 Fig. Probability visualization of noisy test data.** (A) prediction probabilities of original noisy data, (B) predictions of noise reduced data (The number of standard deviations above the noise is set to ‘0.1’, and mode of stationary set to ‘True’), and (C) different setting of noise reduced data (default setting from library). | |

**Comment 5, 6:**
Could you add a link for downloading your approach? Could you add a link for downloading the datasets used in your paper?

**Our response:**
Of course, we will open our GitHub repository to approach our method and data after the paper is completely published (https://github.com/sunghoon-most/Wheeze_Counter).

**References**

1.	Grooby E, Sitaula C, Fattahi D, Sameni R, Tan K, Zhou L, et al. Real-time multi-level neonatal heart and lung sound quality assessment for telehealth applications. IEEE Access. 2022;10:10934-48.

2.	Amodei D, Ananthanarayanan S, Anubhai R, Bai J, Battenberg E, Case C, et al., editors. Deep speech 2: End-to-end speech recognition in english and mandarin. International conference on machine learning; 2016: PMLR.

3.	Stables R, Hockman J, Southall C, editors. Automatic Drum Transcription using Bi-directional Recurrent Neural Networks2016: dblp.

4.	Lim H, Park J-S, Han Y, editors. Rare Sound Event Detection Using 1D Convolutional Recurrent Neural Networks. DCASE; 2017.

5.	Rocha BM, Filos D, Mendes L, Serbes G, Ulukaya S, Kahya YP, et al. An open access database for the evaluation of respiratory sound classification algorithms. Physiological measurement. 2019;40(3):035001.

6.	Pramono RXA, Bowyer S, Rodriguez-Villegas E. Automatic adventitious respiratory sound analysis: A systematic review. PloS one. 2017;12(5):e0177926.

7.	Acharya J, Basu A. Deep neural network for respiratory sound classification in wearable devices enabled by patient specific model tuning. IEEE transactions on biomedical circuits and systems. 2020;14(3):535-44.

8.	Bardou D, Zhang K, Ahmad SM. Lung sounds classification using convolutional neural networks. Artificial Intelligence in Medicine. 2018;88:58-69.

9.	Demir F, Sengur A, Bajaj V. Convolutional neural networks based efficient approach for classification of lung diseases. Health information science and systems. 2020;8(1):1-8.

10.	Demir F, Ismael AM, Sengur A. Classification of lung sounds with CNN model using parallel pooling structure. IEEE Access. 2020;8:105376-83.

11.	Shuvo SB, Ali SN, Swapnil SI, Hasan T, Bhuiyan MIH. A lightweight cnn model for detecting respiratory diseases from lung auscultation sounds using emd-cwt-based hybrid scalogram. IEEE Journal of Biomedical and Health Informatics. 2020;25(7):2595-603.

12.	Perna D, Tagarelli A, editors. Deep auscultation: Predicting respiratory anomalies and diseases via recurrent neural networks. 2019 IEEE 32nd International Symposium on Computer-Based Medical Systems (CBMS); 2019: IEEE.

13.	Kochetov K, Putin E, Balashov M, Filchenkov A, Shalyto A, editors. Noise masking recurrent neural network for respiratory sound classification. International Conference on Artificial Neural Networks; 2018: Springer, Cham.

14.	Hsu FS, Huang SR, Huang CW, Huang CJ, Cheng YR, Chen CC, et al. Benchmarking of eight recurrent neural network variants for breath phase and adventitious sound detection on a self-developed open-access lung sound database. PLoS One. 2021;16(7):e0254134.

15.	Kochetov K, Putin E, Azizov S, Skorobogatov I, Filchenkov A, editors. Wheeze detection using convolutional neural networks. Progress in Artificial Intelligence: 18th EPIA Conference on Artificial Intelligence, EPIA 2017, Porto, Portugal, September 5-8, 2017, Proceedings 18; 2017: Springer.

16.	Rani A, Sehrawat H, editors. Role Of Machine Learning and Random Forest in Accuracy Enhancement During Asthma Prediction. 2022 10th International Conference on Reliability, Infocom Technologies and Optimization (Trends and Future Directions)(ICRITO); 2022: IEEE.

17.	Oletic D, Bilas V. Asthmatic wheeze detection from compressively sensed respiratory sound spectra. IEEE journal of biomedical and health informatics. 2017;22(5):1406-14.

18.	Torre-Cruz J, Canadas-Quesada F, Carabias-Orti J, Vera-Candeas P, Ruiz-Reyes N. A novel wheezing detection approach based on constrained non-negative matrix factorization. Applied Acoustics. 2019;148:276-88.
