# Supplementary material for: Real-time counting of wheezing events from lung sounds using deep learning algorithms: Implications for disease prediction and early intervention
Source: PLoS One. 2023 Nov 20;18(11):e0294447. doi: 10.1371/journal.pone.0294447 (PMC10659186; doi:10.1371/journal.pone.0294447)
Supplement: Attachment — Submitted filename: Response to Reviewers.docx [file pone.0294447.s011.docx]

**Response to Reviewer 2**

**General comments:**
I am satisfied with the revision, and I appreciate the work carried out by the authors to satisfy my comments. I recommend the paper for publication.

**Our response:**
We appreciate your insightful comments, and we would like to express our gratitude to your time and attention in helping us to enhance the quality of our work.

**Response to Reviewer 3**

**General comments:**
The addition of the ICBHI 2017 challenge datasets, adoption of the 10-fold cross-validation method and testing the algorithm's robustness in noisy environments were especially appreciated.

**Our response:**
We appreciate your insightful comments, and we would like to express our gratitude to your time and attention in helping us to enhance the quality of our work.

**Response to Reviewer 4**

**General comments:**
The paper proposes to use the 1d-cnn to count the wheeze events from lung sound dataset.
The problem of study is interesting and relevant.
However, the paper requires several issues to be fixed for the publication.

**Our response:**
We appreciate you to accepting the definition of the issues. We spent plenty of time debating how to respond to your insightful comments, and we responded by highlighting each one with blue. Additionally, we highlighted our revised manuscript with red. We hope that our responses sufficiently satisfy your comments.

**Comment 1**:
Novelty, number of samples tested, datasets used, result metrics etc must be specified in the abstract.

**Our response:**
We thank for your valuable comments. We completely modified the abstract according to the comments and specified novelty, number of samples tested, datasets used, result metrics etc.

| **Before:**  For self-symptom management or remote monitoring via telemedicine, the long-term pattern and frequency of abnormal lung sounds must be monitored and analyzed based on real-time data collection and an automated classification algorithm. To do this, the algorithm is necessary to detect abnormal sounds at each tidal breath as well as the location, duration, and relationships of an entire respiration cycle, including atypical breathing activities. Despite the existence of numerous machine learning algorithms implemented for lung sounds to distinguish abnormal breathing sounds, most lung sound classification methods have not been widely used in practice because of the difficulty of applying them to continuous data. In this exploratory study, we developed a real-time adventitious sound (particularly wheeze) counting algorithm that could be utilized to monitor relatively long-term lung sounds based on a one-dimensional convolutional neural network and a long short-term memory combined (1D-CNN-LSTM) network model. This counting algorithm determines the isolated respiration cycle and identifies and counts the occurrences of abnormal sound during the entire respiration, presenting them in real-time. This real-time wheeze counter could upgrade existing studies that aim to predict various lung diseases based on long-term breathing patterns in both clinical and non-clinical settings for the early detection or remote intervention of exacerbated respiratory symptoms. | **After:**  This pioneering study aims to revolutionize self-symptom management and telemedicine-based remote monitoring through the development of a real-time wheeze counting algorithm. Leveraging a novel approach that includes the detailed labeling of one breathing cycle into three types: break, normal, and wheeze, this study not only identifies abnormal sounds within each breath but also captures comprehensive data on their location, duration, and relationships within entire respiratory cycles, including atypical patterns. This innovative strategy is based on a combination of a one-dimensional convolutional neural network (1D-CNN) and a long short-term memory (LSTM) network model, enabling real-time analysis of respiratory sounds. Notably, it stands out for its capacity to handle continuous data, distinguishing it from conventional lung sound classification algorithms. The study utilizes a substantial dataset consisting of 535 respiration cycles from diverse sources, including the Child Sim Lung Sound Simulator, EMTprep Open-Source Database, Clinical Patient Records, and the ICBHI 2017 Challenge Database. Achieving a classification accuracy of 90%, the exceptional result metrics encompass the identification of each breath cycle and simultaneous detection of the abnormal sound, enabling the real-time wheeze counting of all respirations. This innovative wheeze counter holds the promise of revolutionizing research on predicting lung diseases based on long-term breathing patterns and offers applicability in clinical and non-clinical settings for on-the-go detection and remote intervention of exacerbated respiratory symptoms. |
| --- | --- |

**Comment 2**:
Sufficient novelty is to be established.

**Our response:**
We appreciate your valuable comment. Our paper presents a cutting-edge segmentation algorithm that identifies and counts abnormal respiratory events, aiding in the diagnosis and monitoring of lung diseases. This innovative method, which uses a segment-based supervised learning approach and an AI-based classifier, is trained on three types of labeled lung sound data and focuses on long-term breathing patterns, enhancing its potential for early diagnosis and remote treatment of respiratory diseases.

| **Before:**  Considering this loophole, in this exploratory study, we used relatively long breathing data to develop a real-time event counting algorithm that first determines the respiratory unit, classifies breathing sound anomalies such as wheeze within the unit, records the frequency and pattern of abnormal breathing sound within a certain period, and presents them in real time. We implemented a segment-based supervised learning approach with an AI-based classifier using the predicted probabilities of each segment. | **After:**  Considering this loophole, in this exploratory study, we have developed a real-time event counting algorithm to identify abnormal breathing sounds, especially wheezing, and record their frequency to determine the pattern over a certain period and present this information in real-time. We utilize a unique method that involves the meticulous categorization of a single breathing cycle into three types: break, normal, and wheeze. The algorithm not only detects abnormal sounds in each breath but also collects extensive data on their location, duration, and connections within the entire respiratory cycles, including unusual patterns. |
| --- | --- |

**Comment 3**:
Conclusion section could not be found.

**Our response:**
Thank you for your valuable comment. We have incorporated some of the discussion and added content to the Conclusion section. This not only succinctly conveys the main points of the text but also includes how this research will impact other studies.

| **Before:**  No Text in the original manuscript. | **After:**  **Conclusion**  This research presents a deep learning-based algorithm for counting wheezes, utilizing a 1D-CNN-LSTM model. The model is trained on a variety of reference lung sound databases to predict the probability of abnormal sounds in each segment. Our algorithm then uses this model to count wheeze instances from recorded lung sounds (Algorithm 1) and validates in real-time lung sound simulation (Algorithm 2).  Our wheeze counting method is straightforward yet effective, with potential for expansion into automatic symptom monitoring. This could be crucial in predicting the onset or severity of future abnormalities, as well as detecting current symptoms. Given the possible link between wheeze occurrence trends and symptom exacerbations, our approach could aid in preventing urgent emergencies like asthma attacks. Unlike traditional lung sound classification algorithms, our method can handle continuous data. With a detection accuracy of 90%, the results include identifying the number of total breath cycles and the proportion of abnormal sounds, along with real-time counting and visualization of these events throughout whole respiration. This could revolutionize research on predicting lung diseases based on long-term breathing patterns and offers utility in both clinical and non-clinical settings for immediate detection and remote intervention of worsened respiratory symptoms. Moreover, our counting algorithm can easily adapt to other bio-signals. For instance, when used with ECG (Electrocardiogram) or EMG (Electromyography) signals, it could automatically detect the intensity of heart or muscle anomaly patterns.  In conclusion, our study introduces a novel and effective approach to real-time wheeze detection and counting, which has significant potential for improving self-symptom management and telemedicine-based remote monitoring. This innovative wheeze counter, with its high detection accuracy and ability to handle continuous data, could play a crucial role in predicting lung diseases based on long-term breathing patterns. Furthermore, its adaptability to other bio-signals suggests a wide range of potential applications in both clinical and non-clinical settings. Future research should focus on further refining the algorithm and exploring its potential in various healthcare contexts. |
| --- | --- |

**Comment 4**:
What is the training and testing ratio? Data split ratio is done on what basis?

**Our response:**
We thankful for your valuable comment. In our model, we didn't follow the traditional train-test split ratio. Instead, we used the k-fold cross-validation method which divides the entire dataset into 10 equal parts or 'folds'. Each fold serves as the testing set once, and the remaining 9 folds serve as the training set. So, the data split ratio varies in each iteration of the training and testing process. This method ensures that every data point gets tested at least once and helps in minimizing bias. The use of 10-folds is not arbitrary but is a common practice in machine learning. It is a good balance between computational cost and model performance. If we increase the number of folds, the variance of the resulting estimate decreases, but the bias increases. Conversely, if we decrease the number of folds, the bias decreases, but the variance increases. Therefore, 10-folds is often chosen as it provides a good trade-off between bias and variance.

| **Before:**  The hard-labeled dataset was divided into train and validation data. We used the 10-fold cross-validation method. We utilized the model with the highest accuracy from among the trained models. | **After:**  The hard-labeled dataset was divided into train and validation data. We used the 10-fold cross-validation method. The k-fold cross-validation method is beneficial as it ensures every data point is tested at least once and minimizes bias, providing a more accurate measure of the model's performance. This method also helps mitigate overfitting by using most of the data for fitting and testing each data point at least once. We utilized the model with the highest accuracy from among the trained models. |
| --- | --- |

**Comment 5**:
How do the authors mitigate overfitting?

**Our response:**
Thank you for your comment. We used k-fold cross-validation method for our model validation, which does not follow the traditional train-test split ratio. Instead, it divides the entire dataset into 10 equal parts or 'folds'.

In this method, the model is trained and tested 10 times, each time using a different fold as the testing set and the remaining 9 folds as the training set. This way, each data point gets to be in the testing set exactly once and in the training set 9 times. This process helps in minimizing the bias as we are using most of the data for fitting, and also, we are ensuring that every data point gets tested at least once. The advantage of using k-fold cross-validation is that all the entries in the original dataset are used for both training and testing. This provides a more accurate measure of how well the model generalizes to unseen data, which is the ultimate goal of a machine learning model. The choice of the number of folds depends on the size of the dataset. In our case, we chose 10 folds, which is a common choice in machine learning, as it offers a good trade-off between computational cost and model performance.

| **Before:**  The hard-labeled dataset was divided into train and validation data. We used the 10-fold cross-validation method. We utilized the model with the highest accuracy from among the trained models. | **After:**  The hard-labeled dataset was divided into train and validation data. We used the 10-fold cross-validation method. The k-fold cross-validation method is beneficial as it ensures every data point is tested at least once and minimizes bias, providing a more accurate measure of the model's performance. This method also helps mitigate overfitting by using most of the data for fitting and testing each data point at least once. We utilized the model with the highest accuracy from among the trained models. |
| --- | --- |

**Comment 6**:
How to validate the model?

**Our response:**
Model validation is a crucial step in the machine learning process to ensure that your model has learned effectively from the training data and can generalize well to new, unseen data.

In our case, we employed the k-fold cross-validation technique for validating our model. This method works by dividing the entire dataset into 'k' equal segments or 'folds'. For example, if k equals 10, the dataset is split into 10 equal parts. The model is subsequently trained and tested 'k' times. During each cycle, one of the 'k' subsets is utilized as the test set, while the remaining 'k-1' subsets are combined to create a training set. The performance of the model is then averaged across the 'k' cycles to offer a more reliable measure of its effectiveness. This technique ensures that every data point in the dataset is used for both training and testing at least once, offering a thorough evaluation of the model's performance.

| **Before:**  The hard-labeled dataset was divided into train and validation data. We used the 10-fold cross-validation method. We utilized the model with the highest accuracy from among the trained models. | **After:**  The hard-labeled dataset was divided into train and validation data. We used the 10-fold cross-validation method. The k-fold cross-validation method is beneficial as it ensures every data point is tested at least once and minimizes bias, providing a more accurate measure of the model's performance. This method also helps mitigate overfitting by using most of the data for fitting and testing each data point at least once. We utilized the model with the highest accuracy from among the trained models. |
| --- | --- |

**Comment 7**:
Comparison with similar methods would be beneficial.

**Our response:**
Thank you for your kind comment. We have compiled the research trends in lung sound analysis and related papers into a single table. The table has been added to the supplementary materials.

| Methodology | Results |
| --- | --- |
| Neural Networks [1] | Assists in automatic detection and classification of adventitious lung sounds. |
| Classifiers [2, 3] | Used for automatic detection and classification of lung sounds. |
| Non-negative Matrix Factorization (NMF) [4] | Assists in automatic detection and classification of adventitious lung sounds. |
| Deep Learning Algorithms [5] | Trains the machine to automatically learn the characteristics of the signals or waveforms of lung sounds. |
| Convolutional Neural Network (CNN) [6-11] | Extracts breathing sound features from a two-dimensional spectrogram image. Accuracy ranges from 63% to 99%. CNN-based model generally has the highest accuracy. |
| Recurrent Neural Network (RNN) Model [12, 13] | Used for lung sound classification. |
| Convolutional-Recurrent Neural Network (CRNN) [5, 14] | Combination of CNN and RNN for lung sound classification. |
| AI-based Lung Sound Analysis [15, 16] | Suggested for determining the degree of airway inflammation or risk of a number of lung diseases. |
| Collection of Breathing Sounds from Smartphones or Real-time Lung Sounds from Wearable Devices [17-22] | Used to develop automated AI-based solutions for lung sound analysis and classification. Through this technological advancement, abnormal respiratory and asthmatic symptoms could be detected or diagnosed at an early stage via real-time self-monitoring or telemedicine. |
| Our study: 1D-CNN and LSTM Network Model | Achieved a detection accuracy of 90%. Not only identifies abnormal sounds within each breath but also captures comprehensive data on their location, duration, and relationships within entire respiratory cycles. Capable of handling continuous data. Utilizes a substantial dataset consisting of 535 respiration cycles from diverse sources. |

| **Before:**  The real-time data collection and automated pre-processing system would be critical for long-term monitoring and intervention [23]. | **After:**  The real-time data collection and automated pre-processing system would be critical for long-term monitoring and intervention [23]. We have summarized the relevant papers in a table and included it in S1 Table. |
| --- | --- |

**Comment 8**:
Implications from the survey, research gaps identified, how this work addresses those must be spelled clearly.

**Our response:**
Thank you for your positive comment. We provide specific explanations of the implications derived from the survey, the research gaps identified, and how this work addresses those issues. These details have been incorporated into the main text's introduction.

Implications:

The implications of this research are significant for the field of respiratory medicine. This study demonstrates the potential of AI-based technology in diagnosing and monitoring lung diseases. The development of a real-time event counting algorithm that can identify and classify abnormal breathing sounds, record their frequency and pattern over a specific period, and present this information in real-time, could revolutionize the way lung diseases are diagnosed and monitored. This could lead to earlier detection of lung diseases, more effective treatment plans, and improved patient outcomes.

Research Gaps:

Despite the advancements in AI-based lung sound analysis, there are still several research gaps that need to be addressed. Most existing models focus on the automatic diagnosis of a single recorded data, and applications to real-time monitoring data are still limited. They are developed based on short-term learning data and are not adaptable for real-time, continuous long-term signals. Additionally, most models focus on detecting the presence or absence of abnormalities at each respiratory unit, rather than analyzing the pattern and frequency of abnormal lung sounds over a longer period.

Addressing the Gaps:

This study addresses these research gaps by developing a real-time event counting algorithm that can identify and classify abnormal breathing sounds, record their frequency and pattern over a specific period, and present this information in real-time. The algorithm is designed to be adaptable for real-time, continuous long-term signals, and focuses on analyzing the pattern and frequency of abnormal lung sounds over a longer period, rather than simply detecting the presence or absence of abnormalities at each respiratory unit. This represents a significant advancement in the field of AI-based lung sound analysis, and could have important implications for the diagnosis and monitoring of lung diseases.

| **Before:**  Using three types of labeled lung sound data, we trained a one-dimensional convolutional neural network and a long short-term memory (1D-CNN-LSTM) network model for discriminating three breathing statuses (break, normal, and wheezing) and then developed a “real-time wheezing counter” as a pilot; we suggested the possibility of its application for early diagnosis or the remote treatment of respiratory diseases. | **After:**  Using three types of labeled lung sound data, we trained a one-dimensional convolutional neural network and a long short-term memory (1D-CNN-LSTM) network model for discriminating three breathing statuses (break, normal, and wheezing) and then developed a “real-time wheezing counter” as a pilot; we suggested the possibility of its application for early diagnosis or the remote treatment of respiratory diseases. Our research demonstrates the potential of AI-based technology for diagnosing and monitoring lung diseases in real-time, offering the prospect of earlier detection and improved treatment outcomes. Existing research gaps include limitations in real-time applications and a focus on short-term data. We address these gaps with a real-time event counting algorithm designed for continuous, long-term signals, emphasizing the pattern and frequency of abnormal lung sounds over time, rather than just detecting their presence or absence at individual respiratory units. This advancement holds promise for enhancing the diagnosis and monitoring of lung diseases. |
| --- | --- |

**Comment 9**:
Need content reorganization.

**Our response:**
We appreciate your kind comment. We have moved the Model structure and Clinical lung sound data in this study from the Method section to the Results section and added a Conclusion and a Acknowledgements section.

| **Before:**  Abstract  Introduction  Methods  Clinical lung sound data in this study  Overview  Lung sound databases  Soft labeling and data augmentation  Hard labeling and feature extraction  Model structure  Results  Model training and validation  Validation through visualizing predicted probabilities  Counting algorithm  Application to clinical data  Discussion  Code availability | **After:**  Abstract  Introduction  Methods  Overview  Clinical lung sound data in this study  Lung sound databases  Soft labeling and data augmentation  Hard labeling and feature extraction  Results  Model structure  Model training and validation  Validation through visualizing predicted probabilities  Counting algorithm  Application to clinical data  Discussion  Conclusion  Code availability  Acknowledgements |
| --- | --- |

**Comment 10**:
Weak discussion.

**Our response:**
We’re thankful for your valuable comment. We have supplemented the weak discussion by adding more content.

| **Before:**  In general, automated long-term monitoring via AI-based algorithms could assist preventative medicine by acquiring precursory information from numerous signals and images from the human body for the relevant bad health impacts. As so, long-term monitoring of wheezing occurrences and patterns may shed light on the development of various respiratory illness outcomes if combined with a patient's clinical records, such as symptom exacerbation and response to treatment. | **After:**  In general, automated long-term monitoring via AI-based algorithms could assist preventative medicine by acquiring precursory information from numerous signals and images from the human body for the relevant bad health impacts. As so, long-term monitoring of wheezing occurrences and patterns may shed light on the development of various respiratory illness outcomes if combined with a patient's clinical records, such as symptom exacerbation and response to treatment. The integration of AI-based algorithms into long-term monitoring could revolutionize preventative medicine. By acquiring precursory information from numerous signals and images from the human body, AI could potentially predict adverse health impacts. In the context of respiratory health, long-term monitoring of wheezing occurrences and patterns, combined with a patient's clinical records, could provide invaluable insights into the development of various respiratory illness outcomes. This could lead to more personalized treatment plans and improved patient outcomes. |
| --- | --- |

**Response to Reviewer 5**

**General comments:**
Please proofread the paper before it can be accepted. Please recheck the format of the refences and citations.

**Our response:**
We appreciate your valuable comments. As your suggested, we rechecked our manuscript before resubmitting. We would like to express our gratitude to your time and attention in helping us to enhance the quality of our work.

**References**

1.	Kochetov K, Putin E, Azizov S, Skorobogatov I, Filchenkov A, editors. Wheeze detection using convolutional neural networks. Progress in Artificial Intelligence: 18th EPIA Conference on Artificial Intelligence, EPIA 2017, Porto, Portugal, September 5-8, 2017, Proceedings 18; 2017: Springer.

2.	Rani A, Sehrawat H, editors. Role Of Machine Learning and Random Forest in Accuracy Enhancement During Asthma Prediction. 2022 10th International Conference on Reliability, Infocom Technologies and Optimization (Trends and Future Directions)(ICRITO); 2022: IEEE.

3.	Oletic D, Bilas V. Asthmatic wheeze detection from compressively sensed respiratory sound spectra. IEEE journal of biomedical and health informatics. 2017;22(5):1406-14.

4.	Torre-Cruz J, Canadas-Quesada F, Carabias-Orti J, Vera-Candeas P, Ruiz-Reyes N. A novel wheezing detection approach based on constrained non-negative matrix factorization. Applied Acoustics. 2019;148:276-88.

5.	Acharya J, Basu A. Deep neural network for respiratory sound classification in wearable devices enabled by patient specific model tuning. IEEE transactions on biomedical circuits and systems. 2020;14(3):535-44.

6.	Aykanat M, Kilic O, Kurt B, Saryal S. Classification of lung sounds using convolutional neural networks. EURASIP Journal on Image and Video Processing. 2017;2017(1):1-9.

7.	Bardou D, Zhang K, Ahmad SM. Lung sounds classification using convolutional neural networks. Artificial Intelligence in Medicine. 2018;88:58-69.

8.	Demir F, Ismael AM, Sengur A. Classification of lung sounds with CNN model using parallel pooling structure. IEEE Access. 2020;8:105376-83.

9.	Demir F, Sengur A, Bajaj V. Convolutional neural networks based efficient approach for classification of lung diseases. Health information science and systems. 2020;8(1):1-8.

10.	Shuvo SB, Ali SN, Swapnil SI, Hasan T, Bhuiyan MIH. A lightweight cnn model for detecting respiratory diseases from lung auscultation sounds using emd-cwt-based hybrid scalogram. IEEE Journal of Biomedical and Health Informatics. 2020;25(7):2595-603.

11.	Tariq Z, Shah SK, Lee Y. Feature-based Fusion using CNN for Lung and Heart Sound Classification. Sensors. 2022;22(4):1521.

12.	Kochetov K, Putin E, Balashov M, Filchenkov A, Shalyto A, editors. Noise masking recurrent neural network for respiratory sound classification. International Conference on Artificial Neural Networks; 2018: Springer, Cham.

13.	Perna D, Tagarelli A, editors. Deep auscultation: Predicting respiratory anomalies and diseases via recurrent neural networks. 2019 IEEE 32nd International Symposium on Computer-Based Medical Systems (CBMS); 2019: IEEE.

14.	Hsu FS, Huang SR, Huang CW, Huang CJ, Cheng YR, Chen CC, et al. Benchmarking of eight recurrent neural network variants for breath phase and adventitious sound detection on a self-developed open-access lung sound database. PLoS One. 2021;16(7):e0254134.

15.	Shimoda T, Obase Y, Nagasaka Y, Nakano H, Ishimatsu A, Kishikawa R, et al. Lung sound analysis helps localize airway inflammation in patients with bronchial asthma. Journal of Asthma and Allergy. 2017;10:99-108.

16.	Aziz S, Khan MU, Shakeel M, Mushtaq Z, Khan AZ. An Automated System towards Diagnosis of Pneumonia using Pulmonary Auscultations. 2019 13th International Conference on Mathematics, Actuarial Science, Computer Science and Statistics (MACS)2019. p. 1-7, doi: 10.1109/MACS48846.2019.9024789.

17.	Rocha BM, Filos D, Mendes L, Serbes G, Ulukaya S, Kahya YP, et al. An open access database for the evaluation of respiratory sound classification algorithms. Physiological Measurement. 2019;40(3):035001.

18.	Faezipour M, Abuzneid A. Smartphone-based self-testing of COVID-19 using breathing sounds. Telemedicine and e-Health. 2020;26(10):1202-5.

19.	Gupta P, Moghimi MJ, Jeong Y, Gupta D, Inan OT, Ayazi F. Precision wearable accelerometer contact microphones for longitudinal monitoring of mechano-acoustic cardiopulmonary signals. NPJ Digital Medicine. 2020;3(1):1-8.

20.	Jaber MM, Abd SK, Shakeel PM, Burhanuddin MA, Mohammed MA, Yussof S. A telemedicine tool framework for lung sounds classification using ensemble classifier algorithms. Measurement. 2020;162(107883).

21.	Srivastava A, Jain S, Miranda R, Patil S, Pandya S, Kotecha K. Deep learning based respiratory sound analysis for detection of chronic obstructive pulmonary disease. PeerJ Computer Science. 2021;7:e369.

22.	Lella KK, Pja A. Automatic diagnosis of COVID-19 disease using deep convolutional neural network with multi-feature channel from respiratory sound data: cough, voice, and breath. Alexandria Engineering Journal. 2022;61(2):1319-34.

23.	Naqvi SZH, Choudhry MA. An automated system for classification of chronic obstructive pulmonary disease and pneumonia patients using lung sound analysis. Sensors. 2020;20(22):6512.
